# COVID-SAFE: An IoT-Based System for Automated Health Monitoring and Surveillance in Post-Pandemic Life

**DOI:** 10.1109/ACCESS.2020.3030194

**Published:** 2020-10-12

**Authors:** Seyed Shahim Vedaei, Amir Fotovvat, Mohammad Reza Mohebbian, Gazi M. E. Rahman, Khan A. Wahid, Paul Babyn, Hamid Reza Marateb, Marjan Mansourian, Ramin Sami

**Affiliations:** 1 Department of Electrical and Computer EngineeringUniversity of Saskatchewan 7235 Saskatoon SK S7N 5A9 Canada; 2 College of MedicineSaskatchewan Health Authority 7234 Saskatoon SK S7K 0M7 Canada; 3 Biomedical Engineering DepartmentEngineering FacultyUniversity of Isfahan 48437 Isfahan 8415683111 Iran; 4 Department of Epidemiology and BiostatisticsSchool of HealthIsfahan University of Medical Sciences 48455 Isfahan 8174673461 Iran; 5 Department of Internal MedicineSchool of MedicineIsfahan University of Medical Sciences 48455 Isfahan 8174673461 Iran

**Keywords:** IoT, health monitoring, smart healthcare, pandemic, COVID-19

## Abstract

In the early months of the COVID-19 pandemic with no designated cure or vaccine, the only way to break the infection chain is self-isolation and maintaining the physical distancing. In this article, we present a potential application of the Internet of Things (IoT) in healthcare and physical distance monitoring for pandemic situations. The proposed framework consists of three parts: a lightweight and low-cost IoT node, a smartphone application (app), and fog-based Machine Learning (ML) tools for data analysis and diagnosis. The IoT node tracks health parameters, including body temperature, cough rate, respiratory rate, and blood oxygen saturation, then updates the smartphone app to display the user health conditions. The app notifies the user to maintain a physical distance of 2 m (or 6 ft), which is a key factor in controlling virus spread. In addition, a Fuzzy Mamdani system (running at the fog server) considers the environmental risk and user health conditions to predict the risk of spreading infection in real time. The environmental risk conveys from the virtual zone concept and provides updated information for different places. Two scenarios are considered for the communication between the IoT node and fog server, 4G/5G/WiFi, or LoRa, which can be selected based on environmental constraints. The required energy usage and bandwidth (BW) are compared for various event scenarios. The COVID-SAFE framework can assist in minimizing the coronavirus exposure risk.

## I. Introduction

Internet of Things (IoT) development brings new opportunities in many applications, including smart cities and smart healthcare. Currently, the primary usage of the IoT in healthcare can be categorized as remote monitoring and real-time health systems. Controlling and managing dire situations, such as the one in 2020 when the coronavirus disease (COVID-19) took over the world, can be achieved with the help of IoT systems, without imposing severe restrictions on people and industries. COVID-19 causes respiratory symptoms and appears to be more contagious in comparison to SARS in 2003 [1]. One way to control the spread of viruses, until a vaccine is available, is to observe physical (or social) distancing [2]. By implementing better systems for surveillance, healthcare, and transportation, contagious diseases will have less chance of spreading [3], [4]. An IoT system, combined with Artificial Intelligence (AI), may offer the following contributions when considering a pandemic [5]: 1) improving public security using surveillance and image recognition systems, 2) utilizing drones for supply, delivery, or disinfection, 3) contact tracing and limiting people’s access to public places through apps and platforms empowered with AI. An IoT system for healthcare is typically composed of many sensors connected to a server; it gives real-time monitoring of an environment or users. In a pandemic, AI-assisted sensors can be used to help predict whether or not people are infected with the virus, based on signs such as body temperature, coughing patterns, and blood oxygen levels. Tracking people’s geolocation can be another useful feature. During the outbreak of a contagious disease, tracking the distance between people can provide valuable information. Using technologies, such as Bluetooth, we can get a reasonable estimate of how much distance people maintain when walking in public places. This data can be used to warn people who are not physically distanced within a specific range, 2 m for example [6], of a person, and thereby, potentially prevent further transmission of the virus. During the development of such platforms, it is also crucial to consider security and data management thoroughly to prevent abuse of personal information [7], [8]. Governments may try to use these platforms and information for permanent surveillance after a pandemic to control and track people’s behaviors.

## II. Related Works

During the last several years, different IoT applications have been proposed to improve healthcare systems. The IoT can be used for remote patient monitoring, e.g., connecting seniors who have chronic diseases to doctors and medical resources [9]. IoT applications have been implemented to aid people with Parkinson’s [10] and Alzheimer’s disease [11]. It offers disaster management for seniors who are living alone and need special care [12] and can also be applied to manage equipment and patients in hospitals [13]. In a smart healthcare setting, the IoT can help to provide a remote diagnosis prior to hospitals for more efficient treatment [14]. For diabetic patients, it is vital to monitor their blood glucose continuously [15]; blood glucose data can be sent from wearable sensors to doctors or smartphones for continuous monitoring of patients’ state of health. Castillejo *et al.* [16] develop an IoT e-health system based on Wireless Sensor Networks (WSN) for firefighters.

Geolocation of people gives important information about a potential outbreak during a pandemic. This process can be performed in many ways, each having its pros and cons although providing accurate estimations. A global positioning system (GPS) uses large power consumption. However, GPS accuracy can be severely degraded based on the position of a receiver and satellites, especially indoors [17]. The work in [18] has demonstrated the feasibility of using the Received Signal Strength Indicator (RSSI) to locate the user in an indoor environment. The user carries a mobile which is connected to the Wireless Local Area Network (WLAN). The mobile sends a signal to several fixed position access points (APs), which are then fused using a Center of Gravity algorithm to locate the user. Chawathe [19] conveys the usage of Bluetooth beacons for geolocation tracking. Bluetooth is used everywhere from smartwatches to phones, but one problem of using this technology is the reflection of its signals, which makes it difficult to acquire accurate distance estimations. In [20], a low-power tracking method for IoT systems is proposed. It uses an orientation sensor and accelerometer for geolocation tracking to reduce the use of GPS, which requires less power consumption. Recently, Apple and Google announced that they would be using Bluetooth for contact tracing of iOS and Android users [21]. Users can turn it on or off, and the data would only be given to trusted health authorities that follow specified privacy policies.

Audio signal processing is another area that can be helpful for the diagnosis of many respiratory diseases. For COVID-19, the patients with advanced cases often suffer from coughing in, but it can also be a symptom of influenza and many other medical conditions [22]. Currently, many research groups are working on this idea to battle COVID-19 [23], including Coughvid from Ecole Polytechnique Federale de Lausanne (EPFL) [24], Breath for Science from NYU [25], CoughAgainstCovid from Wadhwani AI group in collaboration with Stanford University [26], and COVID Voice Detector from Carnegie Mellon University [27]. Imran *et al.* [22] have made an AI model to distinguish between coughs related to COVID-19 and coughs coused by other respiratory conditions. Their model has achieved promising results; however, their dataset is not large enough. Providing more data about the coughing of COVID-19 patients will make such AI models much more effective.

FluPhone [28] is one of the first projects that utilized users’ phones to study how fast an infectious disease spreads. Mobile phones were used to collect some data, such as the presence of nearby Bluetooth devices, GPS coordination, and flu symptoms. Then, the data were sent to a server via 3G/GPRS [29]. EpiMap [30] was another project done followed FluPhone. The proposed framework could be used for rural areas or developing countries, where opportunistic networks and satellite communications were employed for the transmission of data. Another recent study [30] evaluates how much active contact tracing and surveillance can reduce the spread of infectious diseases. The results show that mobile phone contact tracing has significant social and economic benefits.

In this article, the proposed COVID-SAFE framework offers: 1) a low-cost and lightweight IoT node to monitor continually a person’s body temperature, heart rate, and blood oxygen saturation, and periodically monitor coughing patterns; 2) a smartphone app to display the parameters and individual risk factors; 3) a physical distance tracking mechanism using Bluetooth 4.0 technology to alert the user in case of violation of safe physical distance; and 4) a fog server that collects data from the IoT nodes and applies a machine-learning algorithm to send the necessary information to users.

## III. Proposed Framework

**FIGURE 1.**
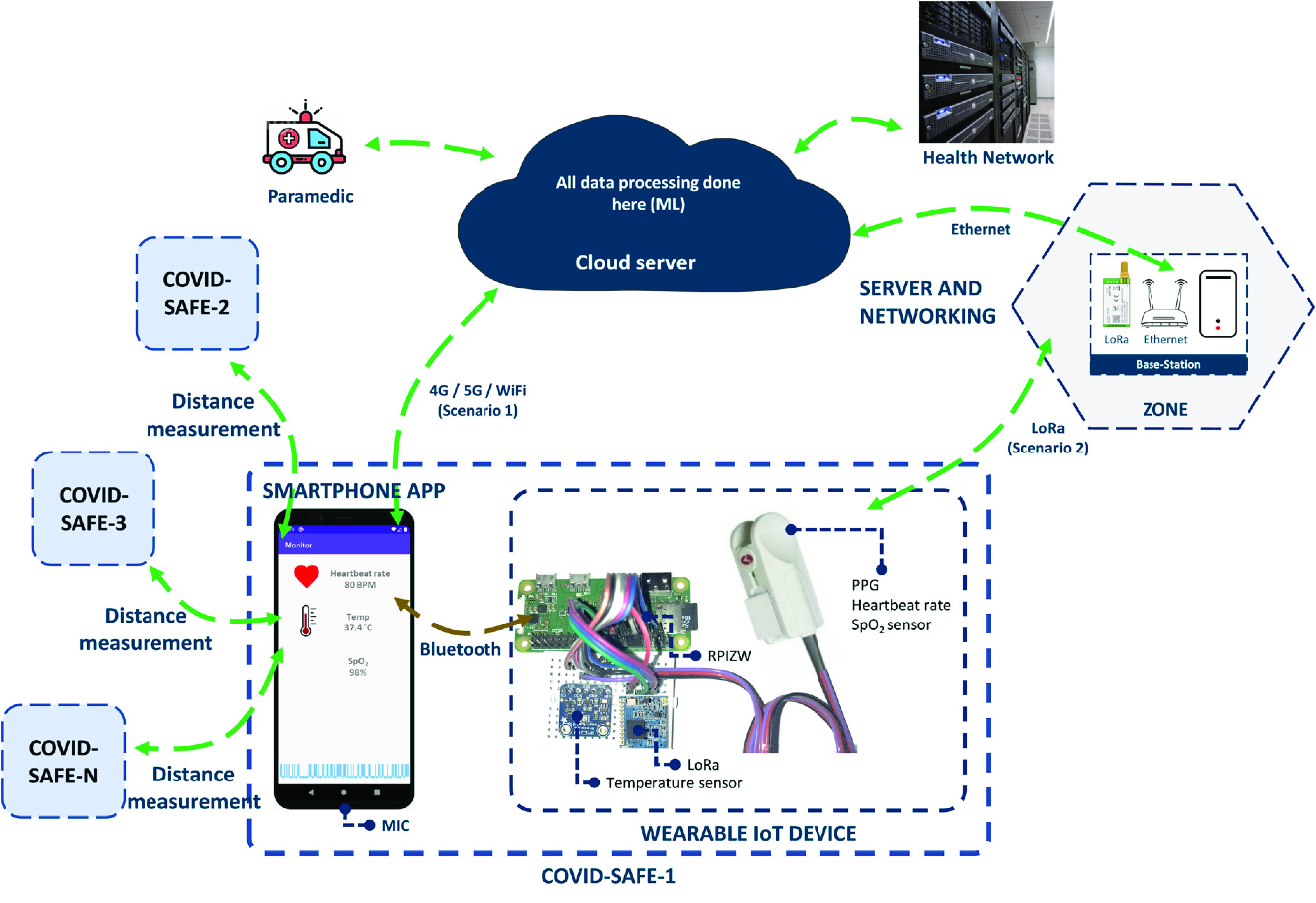
High-level architecture of COVID-SAFE framework, in which COVID-SAFE-1 is carried by the user and COVID-SAFE-2 - N belong to adjacent people.

The development of the COVID-SAFE platform relies on three parts, including a wearable IoT device, smartphone app, and fog (or cloud) server. The hardware contains nodes that were developed on the Raspberry Pi Zero (RPIZ). The software parts include an application program interface (API) for interacting with users on a smartphone, and a fuzzy decision-making system on the fog server. Nodes collect specific vital data from participations and upgrade their decision-making regulations to aid users in various scenarios, such as the need to refer to a doctor, maintaining physical distance from others, and alerts regarding high-risk areas. Fig. 1 illustrates the high-level architecture of the COVID-SAFE framework. A detailed description of each part is given in the next sections.

### A. Wearable IoT Device

This IoT node works in association with the user’s smartphone to collect proximity data using Bluetooth and to communicate with the server through the cellular data network. It consists of a RPIZ as the central processor, temperature and photoplethysmogram sensors, and a LoRa module for data communication in the absence of a cellular data network and WiFi. The system then is synchronized with the software to monitor the user’s behavior during daily activities. In Scenario-1, the IoT node sends the sensor data to the smartphone app via Bluetooth connection. The smartphone then sends the data stream to the server via 4G/5G or WiFi. The server feeds the app with the latest updates. The app can notify users of new restrictions and provide useful tips given by the health service and governments. Meanwhile, the app sends the participations’ body parameters for further processing. The cloud server receives all the information and applies a fuzzy inference system on the data, and finally sends back the risk score to the phone for the user. The second mode of operation (Scenario-2) is a LoRa-based network. The IoT node enters this mode when a 4G/5G/WiFi connection is not available. A possible situation is in rural areas with limited Global System for Mobile Communications (GSM) coverage.

The RPIZ has a 1 GHz single Central Processing Unit (CPU) core with 512 MB of Random Access Memory (RAM), several Global Purpose Input/Outputs (GPIOs), wireless LAN, and Bluetooth connectivity, all in one platform. These features make the RPIZ a suitable choice for implementing many IoT-based systems. The COVID-SAFE framework is equipped with a temperature sensor and a photoplethysmogram (PPG) sensor. The PPG sensor is a noninvasive tool that attaches painlessly to the user’s fingertip, sending two wavelengths of light through the finger, and captures the reflected light using a pin diode. The output of this sensor is a PPG signal. The PPG recording is based on an analog sensor and needs a converter before connecting to the digital part; hence, an analog-to-digital converter (ADC) is used. The RPIZ is equipped with an internal Bluetooth and WiFi module, which makes it easy to interface with a smartphone app. The IoT node is battery operated and is designed with a 3D printer as a finger clip to encapsulate the necessary hardware and to be friendly for the user during daily activities.

In order to measure the power consumption of the system, the wearable IoT device is connected to a digital wattmeter. The data is logged in a computer that produces the wattage measurements.

### B. Smartphone App

**FIGURE 2.**
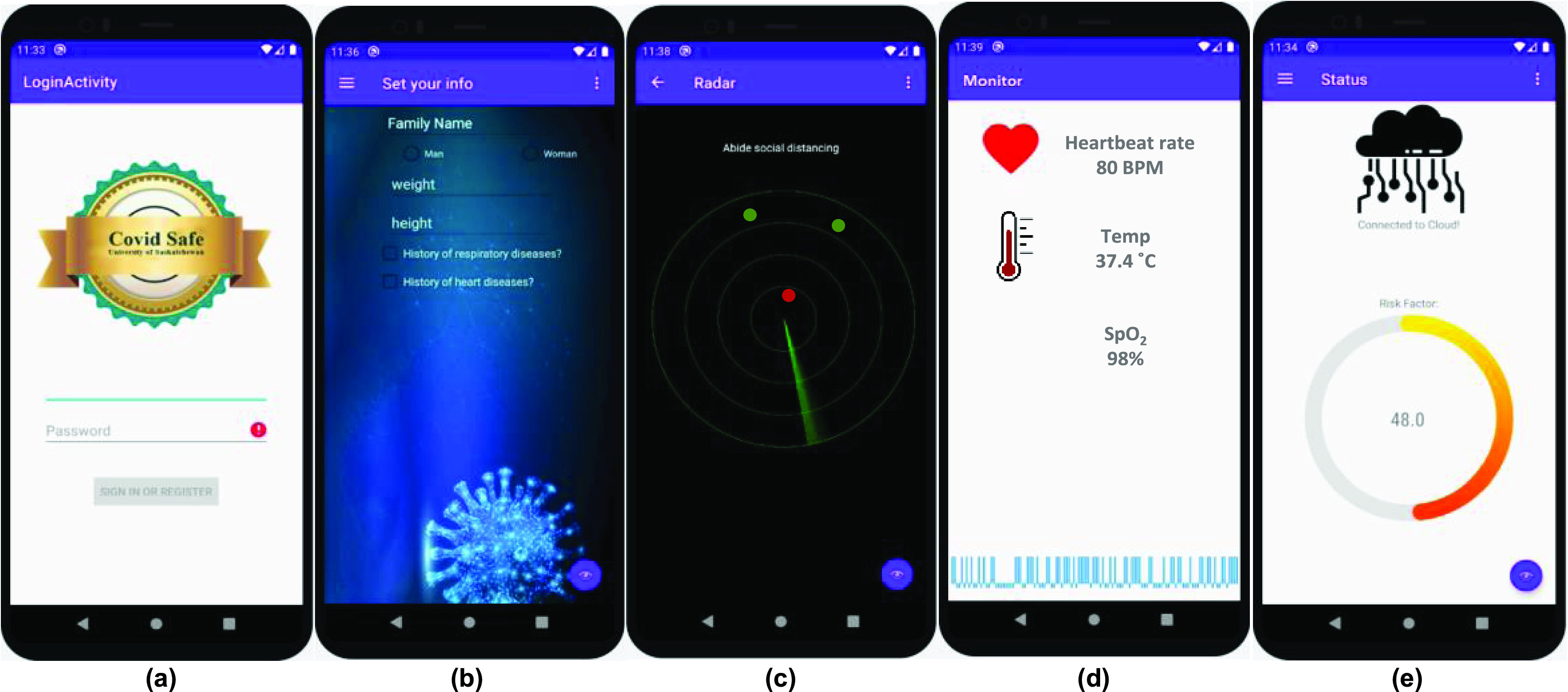
COVID-SAFE application which is connected to fog server based on predefined API, a) login menu b) general information page c) radar dashboard d) health monitoring menu and e) individual risk factor.

Fig. 2 shows the COVID-SAFE smartphone app, which is built to interact easily with users. First, the user has to create an account and answer general background questions such as gender, age, weight, height, and history of diseases. Fig. 2(b) shows the general information page. By accumulating this information, the system can provide an individual risk factor for the user. Fig. 2(c) shows the radar dashboard; in this menu, all adjacent nodes in the range of 3 m are shown on the screen. The red dots illustrate nodes in the range of 2 m or less, the yellow dots indicate nodes between 2 to 3 m, and green dots are nodes placed at 3 m or further. The app notifies the user as soon as the second node comes closer than the specified range. The position of nodes on the radar screen are separated for better visualization purposes. The app can display the heart rate, body temperature, blood oxygen saturation, and individual risk factor in real-time mode as Fig. 2(d) shows. The output of the decision-making system is depicted in Fig. 2(e). In this fragment, the app asks for symptoms following the body parameters, and it provides the risk evaluation, and sends some useful tips.

### C. Decision-Making System

A fuzzy inference system called the decision-making system, is used for predicting the risk of spreading the virus. The model estimates a risk factor containing three linguistic values (low, moderate, and high), which can help users to find out if they are in a safe position or if they might spread a disease. There has been significant evolving activities in this domain that are changing our understanding of symptoms and significant features in diagnosis. For instance, government quarantine strategies and risk tolerance may be changed because of various factors, such as economic circumstances, or factors in different regions of a country. In this regard, a fuzzy decision seems more suitable for predicting the risk factor of a person since it conveys uncertainties. Moreover, all predefined rules in a fuzzy system can be updated regularly based on expert definitions from the cloud. A similar model were developed by other researchers with slightly different input variables [32].

A subset of samples from the Khorshid COVID Cohort (KCC) study [33] was used to design the rules of the proposed decision-making system. Thirty samples from COVID-19 patients (the case group) and thirty other samples from hospitalized pneumonia patients (or patients with similar breathing problems) with negative Polymerase Chain Reaction (PCR) and CT-scan results (the control group) were used in our study. The following baseline patient parameters were considered in the clinical study: gender (female, male), age, body temperature, oxygen saturation (SpO_2_), shortness of breath (yes, no), cough severity (high, increasing-moderate, low), and the presence of chronic respiratory disease (yes, no) (Table 1).

**TABLE 1:** Characteristics of the Participants in the COVID-19 and Non-COVID-19 Groups

	Non-COVID	COVID	$p$-value
Gender	13 (female)	15 (female)	0.873
Age (years)	59.9±19.8	59.5±14.4	0.919
Body temperature	37±1	39±9	0.328
Oxygen saturation (%)	86±8	84±10	0.426
Shortness of breath	9 (no)	9 (no)	0.804
Cough severity	2 (low) 4 (high)	7 (low) 16 (high)	< 0.001
Chronic respiratory disease	18 (no)	27 (no)	0.079
N	30	30	-

MEAN±SD and frequency were provided for continuous and categorical variables, respectively.

In this research, Sugeno architecture [34] is utilized, and an Adaptive-Network-Based Fuzzy Inference System (ANFIS) is used for training memberships and defining rules [35] for simplicity. All membership function types are selected based on a Gaussian function, which is more conventional for training ANFIS. A similar model [36] was developed by other researchers, wherein they selected rules and membership properties manually without using ANFIS. In addition to the ANFIS model, a support vector machine (SVM) [37] and decision tree [38] are trained to be compared with the proposed method. The advantages of a fuzzy system are that it can handle uncertainty and its linguistic rules can be better realized.

The cellphone fetches the rules from the cloud, which is updated regularly. Inputs of the fuzzy system are defined based on health features, and region-based information. Health-related features include respiratory rate, cough rate, temperature, Body Mass Index (BMI), and blood oxygen saturation level. The region-based risk value can be calculated on the server using parameters such as the last time an exposed case was detected and the number of cases in the region.

### D. Data Acquisition

Two different sensors are used in the IoT node. At the startup, the RPIZ initializes all sensors and makes them ready to capture the data. The digital temperature sensor has a 4-byte output resolution. The body temperature usually does not change rapidly; hence, the sensor captures data every 15 or 30 min. In order to have consistency in values, at each iteration, 10 samples are taken, and their average is stored into internal memory storage and also is sent to the server.

The output of the photoplethysmogram sensor is a PPG signal. Due to the nature of the signal, it should be sampled continuously for at least 10 seconds to see the patterns and extract necessary features. The IoT node is responsible to reads the output of the sensor, using an external 8-bit ADC at a 50 Hz sampling rate. By applying the signal processing algorithms on the PPG signal, the heart rate, blood oxygen saturation (SpO_2_), and respiratory rhythm can be extracted [39]. For measuring the SpO_2_ from the signal, first, an average of five subsequent samples of the signals ($A_{1}$ and $A_{2}$) and offsets ($D_{1}$ and $D_{2}$) for red and infrared waveforms (indexed as 1 and 2, respectively) are measured. Then, SpO_2_ is measured using a formula given by Maxim Integrated™.

\begin{equation*} G=-45.060\times K^{2}+30.354\times K+98.845\tag{1}\end{equation*}

where, $K = (A_{1}/D_{1})$ / ($A_{2}/D_{2})$ and $G$ is the SpO_2_ value. According to the literature [40], there is substantial evidence that can increasing respiratory rate is a contributing factor in determining COVID-19. For predicting the respiratory rate from the PPG signal, an adaptive lattice notch filter is utilized based on Park and Lee [41]; the results can achieve 0.78% R-square on the MIMIC II dataset.

This database contains physiological signals and a time series of vital signs captured from patient monitors, as well as comprehensive clinical information obtained from hospital information systems. Furthermore, an average of 10 seconds of an estimated respiratory rate and SpO_2_ are used for reducing prediction error.

The proposed framework can record the surrounding voice using the phone’s microphone to detect the user’s coughing patterns. To save battery power consumption, this feature is activated based on the user’s request. For cough detection, a pre-trained model for acoustic activity prediction is used [42]. For extracting a cough from the environment sounds, a pre-trained model is utilized [43]. After activation, the input microphone is sampled at 5 KHz and an 8-bit resolution for a duration of 10 seconds at each iteration. The reason for choosing 5 KHz is that cough frequency usually accuse between 200–900 Hz [44]. All the sensors’ data are stored in internal memory for further processing.

### E. Proximity Detection

Most of the present smartphones have Bluetooth Low Energy (BLE) V4.0 or above, along with another short-range wireless interface like Near Field Communication (NFC). Table 2 presents the comparison among related wireless technologies. It shows that NFC cannot be used for distance measurement due to its short range, and Bluetooth cannot be used due to its higher power consumption and lack of broadcast capability. On the other hand, using the beacon feature implemented in BLE, a connectionless RSSI monitoring can be used to detect the proximity of the devices or to calculate or measure the relative distance between the smartphones.

**TABLE 2:** Comparison of Smartphone-Based Wireless Interface

	NFC	Bluetooth	Bluetooth Low Energy
RFID compatible	ISO 1 8000-3	Active	Active
Standardization body	ISO/IEC	Bluetooth SIG	Bluetooth SIG
Network Standard	ISO 13157 etc.	IEEE 802.15.1	IEEE 802.15.1
Network Type	Point-to-point	WPAN	WPAN
Cryptography	Not with RFID	available	available
Range	< 0.2 m	~ 10 m (class 2)	~ 100 m
Frequency	13.56 MHz	2.4– 2.5 GHz	2.4– 2.5 GHz
Bit rate	424 Kbit/s	2.1 Mbit/s	~1.0 Mbit/s
Setup time	< 0.1 s	< 6 s	< 0.006 s
Power consumption	< 15 mA (read)	Varies with class	< 15 mA (Tx or Rx)

The proposed method makes it possible to indicate whether another person is located at an adjacent area or not. As soon as the second IoT node (along with the associated phone) comes within range, a flag is raised and the user is notified. The relationship between the transmitted signal strength and received signal power level can be mathematically expressed by equation (2):

\begin{equation*} d={10}^{\left ({\frac {T_{x}-R}{10n} }\right)}\tag{2}\end{equation*}

where, $d$ stands for the distance, $T_{x}$ is the transmit power, $R$ is the received RSSI values, and $n$ is the environmental coefficient.

Two experiments were performed to validate the distance estimation using the RSSI. In the first experiment, two phones are placed at different orientation (face to face and side by side). One phone is placed at a fixed position to record the signal strength, while the second one can move around. In this experiment, the transmit power was set at 4 different levels (−16 dBm, −26 dBm, −35 dBm, and −59 dBm), and the position of the moving phone was changed from 30 to 240 cm with a 30 cm step size. The same experiment was performed again with a 12 cm wooden wall between the scanner phone and broadcasters to consider various orientation and other ambient conditions, such as reflection and absorption.

**FIGURE 3.**
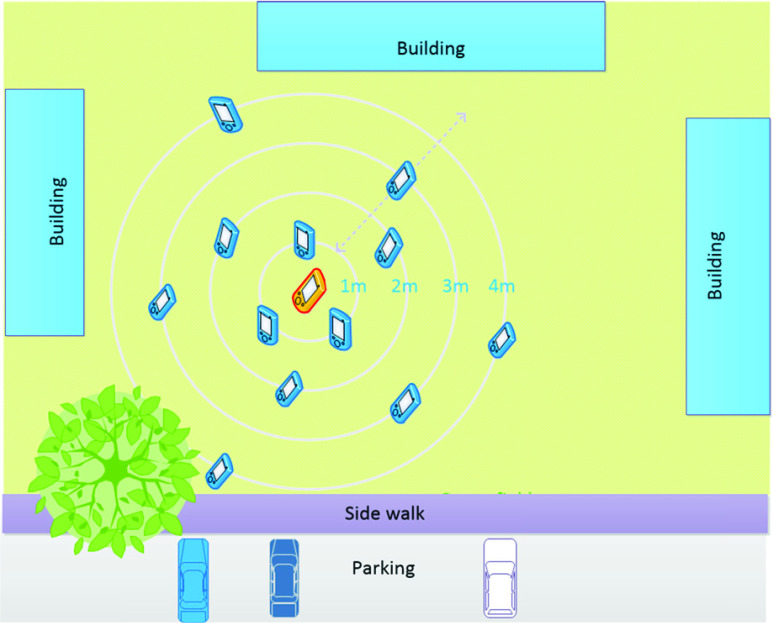
BLE test setup.

In experiment 2, multiple smartphones of different models were used, and Fig. 3 shows a graphical representation of the experimental setup. The RSSI data is acquired in the phone at the center using “Beacon Scanner” with an acquisition frequency of 1 Hz, while other phones are traveling toward and away from the center phone at different angles and orientations. All phones are configured to broadcast the BLE beacon signal (using Google’s Eddystone protocol) at the same interval (3 Hz) with the same transmit power level (−59 dBm). Various angular positions or orientations are defined for the moving phones, and they change their states while the fixed center phone records the received signals.

### F. Server and Networking

All the sensors’ data are sent from the IoT node to the smartphone using WiFi (IEEE 802.11.x standard protocol) as a physical layer for real-time data visualization. Meanwhile, data are transferred to the fog server for further processing. Any transmission of information through the network utilizing IPv4 or IPv6 and the Representational State Transfer (REST) API is given for each participant to access his or her information.

**FIGURE 4.**
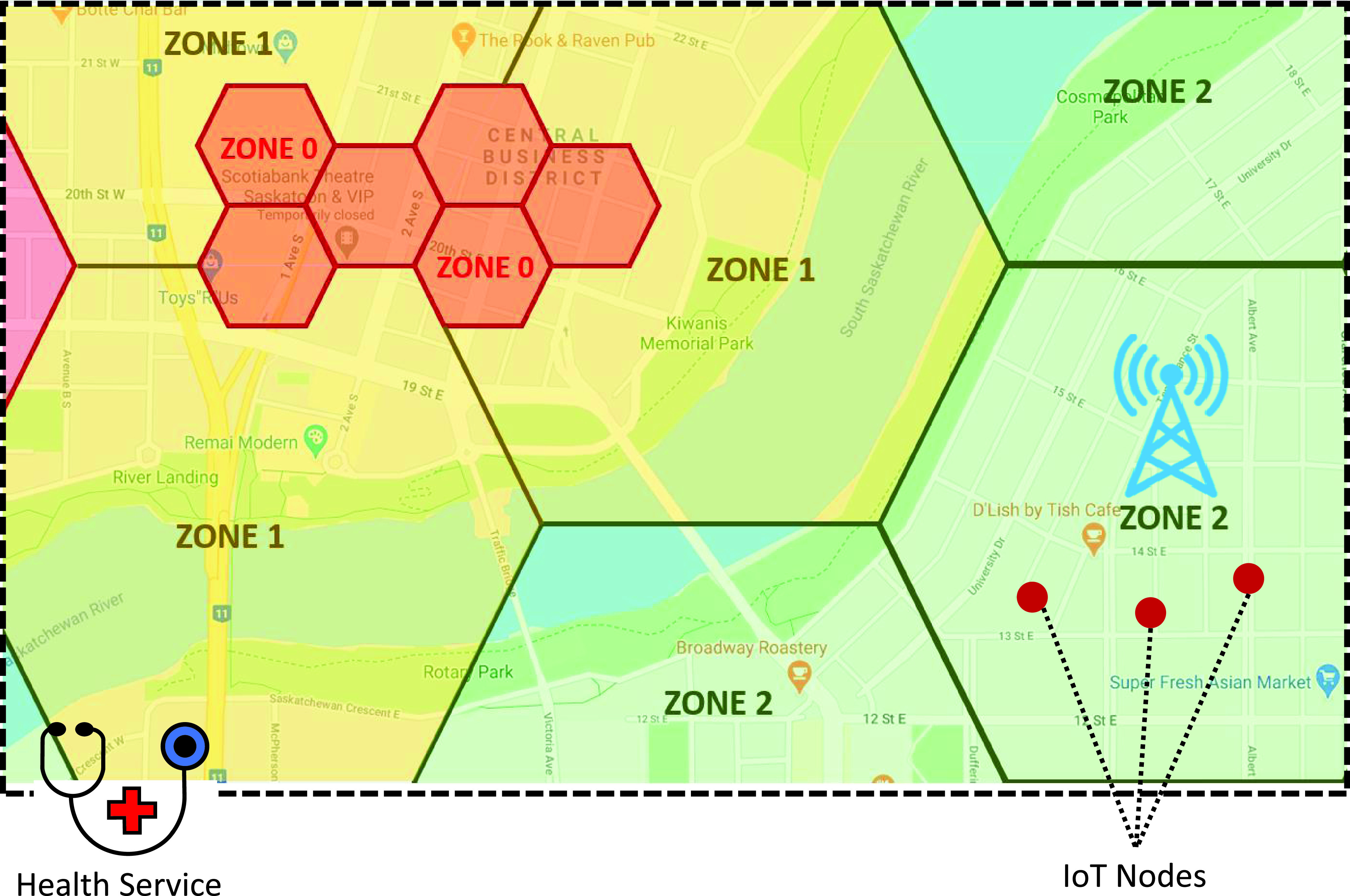
Zone definition (displayed on the smartphone app showing real-time geolocation of hotspots; zone 0 being the most critical with the highest risk of spread; zone 1 has medium risk; zone 2 has low risk).

The main advantage of having REST API is that small devices can use the API even if they have certain limitations such as limited computational capacity and low physical memory. A user can use a designed web page or a smartphone app to link to the services and see his or her status. User data are saved as a user history in the database for potential future development. Connecting to the server can be established either through a 4G/5G infrastructure or LoRa network. Fig. 4 shows a map with different zones; each zone indicates the risk of infection. The database can be updated based on the recent status of regions reported by governments, with parameters such as the number of residences and history of infected people. The map is divided into three colors: green for low risk of infection, yellow for moderate, and red for high.

Zone segmentation has several benefits. First, using the information that each zone provides, users can manage their social activities with the necessary precautions. In addition, governments can send notification to users or limit their access in case of emergency. Thus, the decision-making process is enhanced, and reaction time to a situation is significantly reduced. Information on the zones is then used in risk assessment by the software. The zones should cover the whole map; however, for visualization, only parts of the zones are depicted in Fig. 4.

## IV. Results and Discussion

### A. Distance Measuring

**FIGURE 5.**
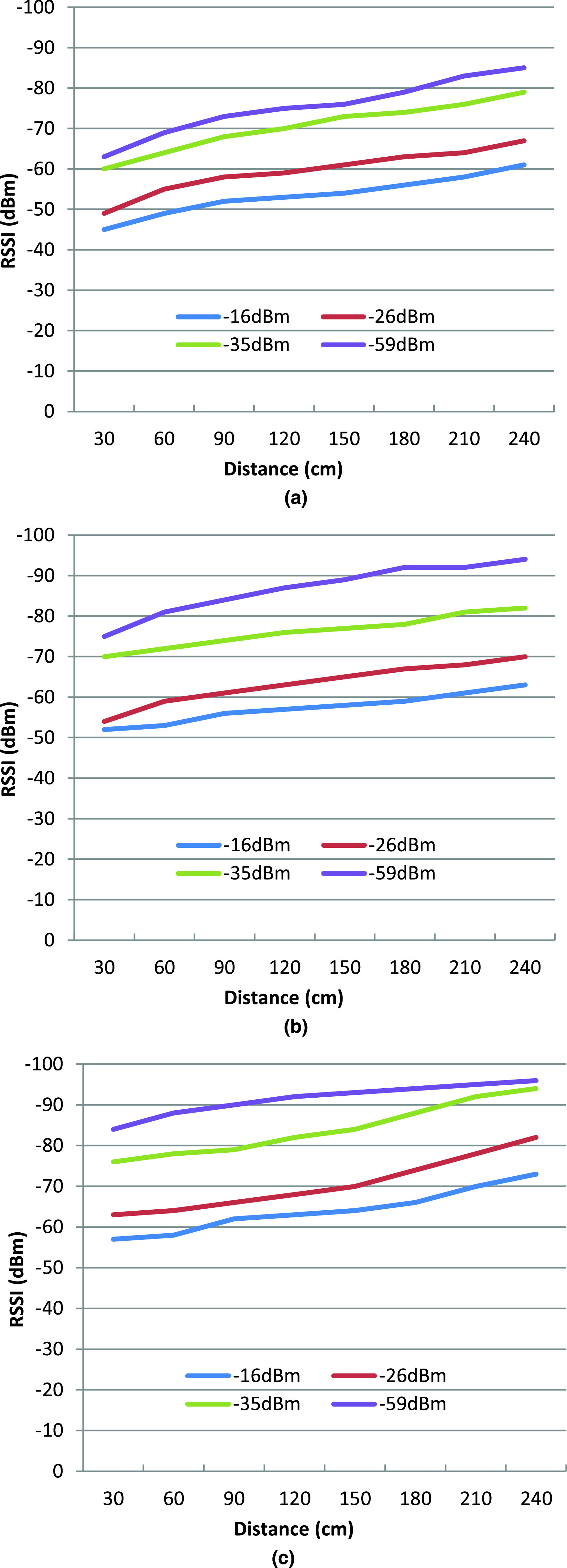
RSSI of BLE for different distances at different T_x_ power gain (dBm), keeping two smartphones in a) face-to-face, b) side-by-side position, and c) two smartphones in the face-to-face position separated by a wooden wall.

According to our experiment, Fig. 5 shows the RSSI values at different distances from 30 to 240 cm, where phones are placed face-to-face, side-by-side, and face to face separated by a wooden wall. The results show that the relative orientation between two IoT nodes could change the RSSI by a maximum value of −10 dBm when phones are placed in side-by-side position. The same experiments were conducted while separating the transmitter and receiver by a wooden wall with a thickness of 10 cm to examine the effect of signal blockage by the wooden wall, and the result is shown in Fig. 5(c). Comparing Fig. 5(a) and 5(b) shows that the RSSI levels depend on the relative positions of the phones. Fig. 5(c) also shows significant changes in the RSSI levels with the presence of a wall in between. As expected, a decrease of RSSI with an increase of distance was observed.

**FIGURE 6.**
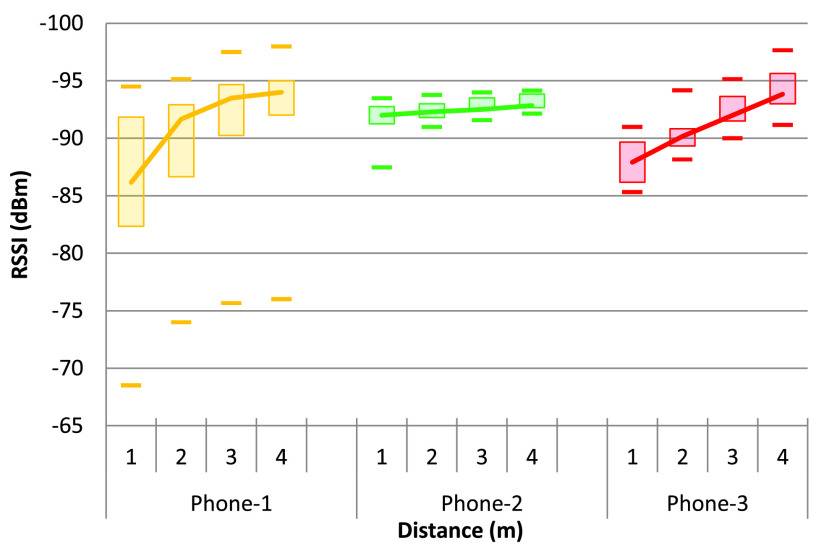
RSSI of BLE at four different distances with various orientation and fixed transmit power for three different types of cellphones.

This result is further justified by experiment 2 (as shown in Fig. 3), and the results are shown in Fig. 6. The data are processed separately for every phone used, and there is a noticeable relationship with the distance from the receiving phone. Although this relationship between RSSI and distance is highly dependent on the device itself (model or hardware construction), this can still be used to calculate the distance between two devices by using Bayesian filters (such as a Kalman filter or particle filter) to reduce the noise in the RSSI data [45].

Equation (2) can be presented in a more straightforward format, as shown in (3), where the environmental coefficient $n$ is replaced by a and $b$. Parameter $b$ is used as a threshold for the initial alarm or to trigger the calculation function locally in the IoT node. Parameter $b$, along with the reference RSSI (RSSI at 1 m distance, denoted as $R_{2}$), is used to calculate the distance from the RSSI measured (denoted as $R_{1}$).

\begin{equation*} {d}={a}{T}_{x}{R}_{1}+{b}+{R}_{2}\tag{3}\end{equation*}

**FIGURE 7.**
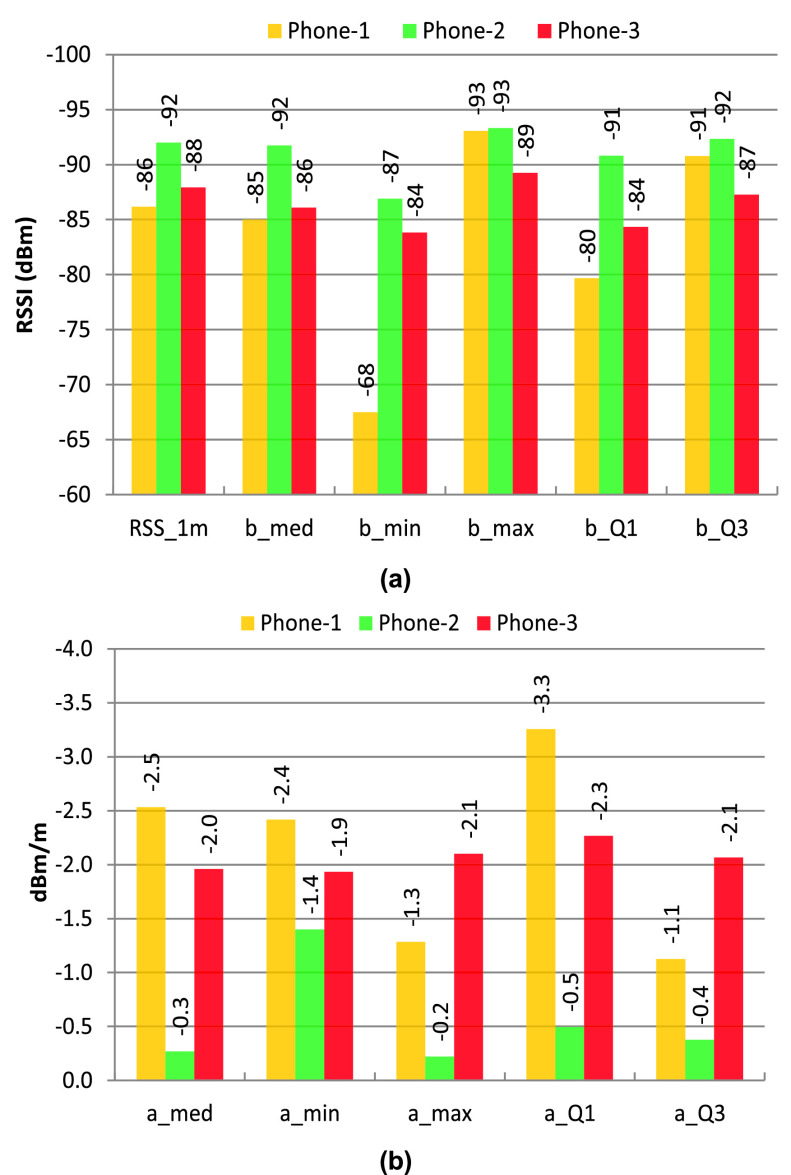
(a) Phone specific RSSI values for the 1 m reference and the RSSI threshold of $b$ for different levels of RSSI data (maximum, minimum, median, Q1 of 75%, and Q3 of 75% RSSI values), (b) phone specific values of a for different levels of RSSI data (maximum, minimum, median, Q1 of 75%, and Q3 of 75%.

Fig. 7(a) shows the phone-specific values of the reference RSSI (at 1 m distance) and the parameter $b$ for different RSSI levels like maximum, minimum, Q1 (lower limit of the 75% quartile), Q3 (upper limit of the 75% quartile); and Fig. 7(b) shows the phone-specific values of the parameter a for different RSSI levels. For this experiment, a threshold of −93 dBm (taken from the value of parameter $b$ for the maximum RSSI level above 2 m distance) can be used to trigger the proximity aware alarm and the distance calculation function in the smartphone app. However, $a$, $b$, and the reference RSSI are dependent on the smartphone used and the real-life environment. There are several algorithms, such as SVM and Machine Learning (ML) [46], with the device or environment-specific training parameterization [47] that can be used to calculate the distance between the devices. In addition, AltBeacon can be used to get device-specific information (manufacturer identification number and 1 m reference RSSI) along with the beacon signal [48] which can be used to improve the distance accuracy for different types of devices used.

In order to notify the user to maintain physical distancing, three threshold values are indicated. The software checks the RSSI values then maps them to the distance according to equation (3). If the distance is less than 200 cm a red flag is raised, if the transmitter is in the range of 200 to 300 cm the flag is yellow, and if the distance is longer than 300 cm it is green.

### B. Decision-Making Results

Table 3 shows the parameters acquired for training the model using ANFIS.

**TABLE 3:** Inputs and Outputs Membership Functions in Proposed Fuzzy System

Feature Name	Description ([$\delta $, $\mu $])
Shortness of breath	Has: [.2,1] Has not: [.2,2]
Cough rate	Normal: [0.35 2.00] Not normal: [0.35 3.00]
Temperature	Cluster 1: [0.70 36.67] Cluster 2: [0.70 37.33] Cluster 3: [0.70 38.00] Cluster 4: [0.70 38.67] Cluster 5: [0.70 39.33]
Age	15 Clusters evenly distributed between 10 and 90 with $\delta =17$
SpO_2_	15 clusters unevenly distributed between 60 and 100 with $\delta =6.7$
Gender	Male: [.2,1] Female: [.2,2]
Predicted risk	15 clusters with Sugeno type

The performance of the proposed method is compared with two ML methods, decision tree and SVM classifiers. The results are provided for five times a training algorithm with shuffling data based on hold-out validation (70% train-30% test) in Table 4.

**TABLE 4:** Accuracy and F1-Score Indices for Test Set According to Different Methods

Method	Accuracy (%)	F1-score (%)
Proposed Method	74.7 ± 4.2	75.3 ± 3.7
Decision tree	72.9 ± 4.0	73.5 ± 3.8
SVM	72.6 ± 4.2	74.1 ± 4.0

**FIGURE 8.**
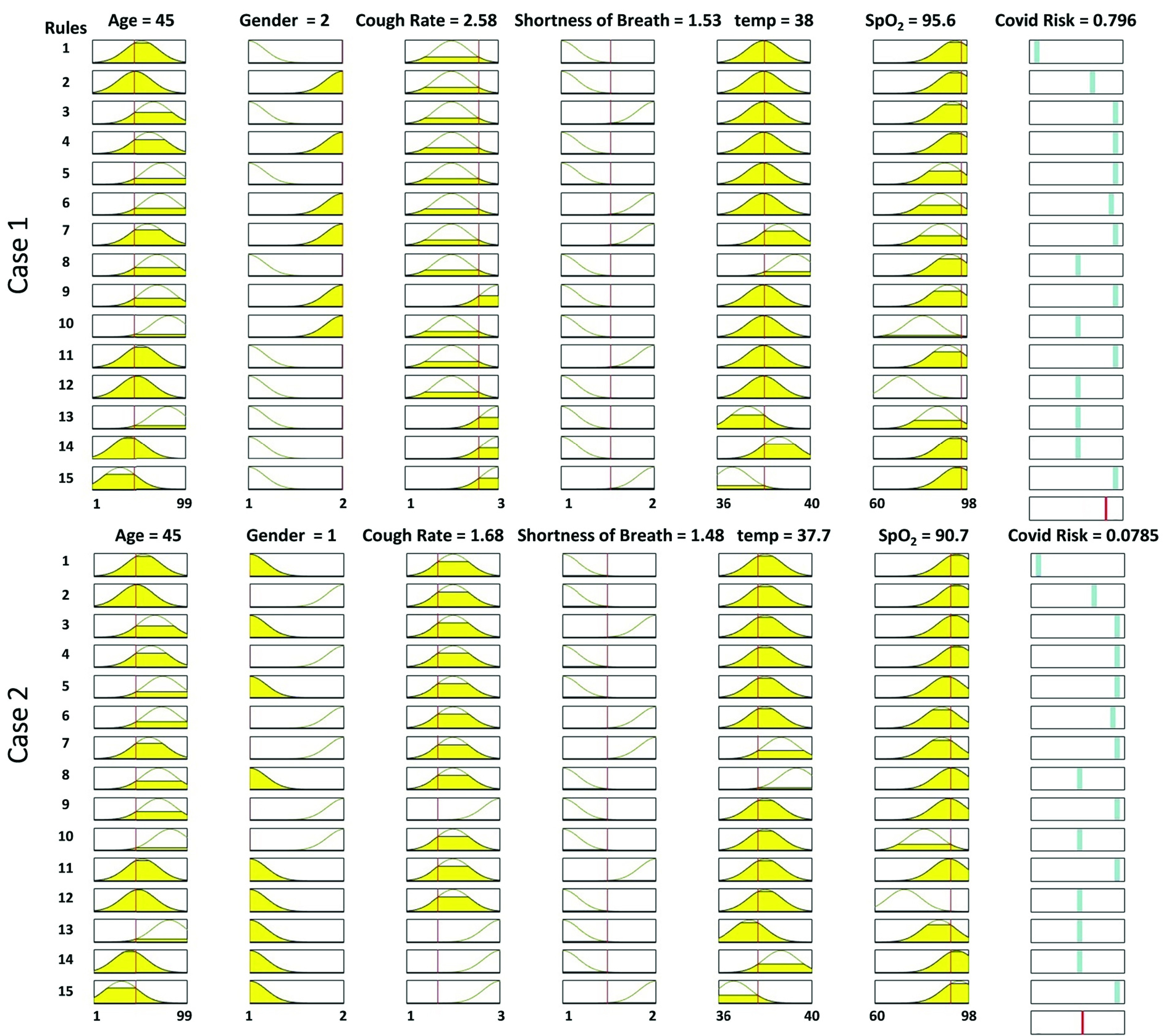
The designed fuzzy inference system based on rules defined in the cloud. Two examples of rules for showing how a person’s risk is generated, based on input features.

Fig. 8 illustrates two examples of fuzzy rules and shows a risk of 0.79 and 0.07 for two people aged 45, with different genders, and similar shortness of breath. The first person has a low fever, and his cough rate is higher than the other persons. It is worth noting that the estimated rules in the fuzzy interference system may not be ideal and can be extended and modified over time based on received feedback. The closed-loop system requires more data and could be addressed in future work.

### C. System Performance

Table. 5 shows scenario-specific activities with power requirements for the various activities measured at the laboratory. According to the measured power, we can quantify the overall energy demand based on scenario-specific activities. Smartphone app power analysis shows that 25 mA is used for all processing in the cellphone. The bandwidth requirement is based on a one-second volume of data generated by the PPG sensors and voice data at the specified sampling rate.

**TABLE 5:** Scenario Specific Activities and Power Requirement

Scenario	No Network	S1	S2	Power consumption (mW)
Data acquisition	Yes	Yes	Yes	678
Data post-processing	Yes	No	Yes	770
LoRa Transfer	No	No	Yes	1040
Cellular network	No	Yes	No	970
BW requirement (bps)		147 K	80	
Data Burst time (sec)		1	0.02	

**FIGURE 9.**
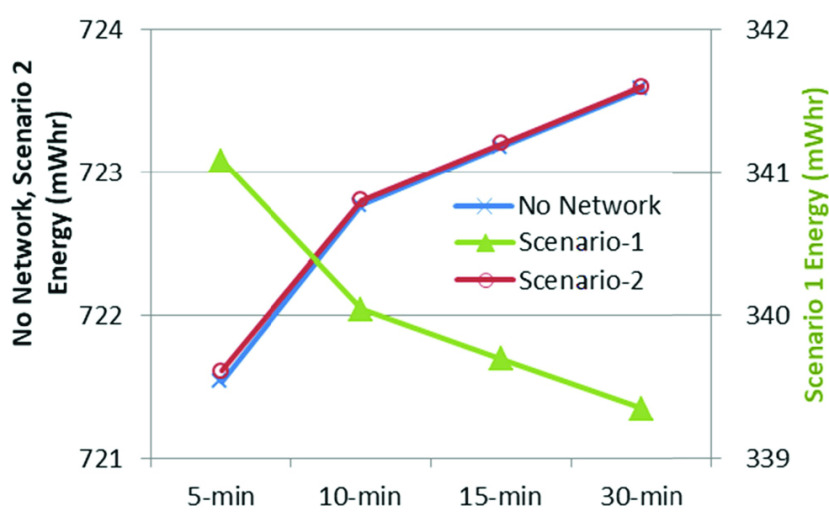
Scenario-specific hourly energy requirement for different transmission intervals.

Time ranges, spanning 5 to 30 minutes, were used for data transfer using LoRa or Bluetooth. Scenario-specific energy demand was distinct, depending on time span. Fig. 9 shows the hourly energy requirement for different transmission intervals; No Network and Scenario-2 require almost the same amount of energy, while Scenario-1 requires less than half of that as there is no offline processing in the IoT node itself. Since the node may need to be carried during only part of the day, the daily energy requirement will also vary depending on the duration of the operation. Scenario-1 is shown on a different scale for better visualization of the changes with transmission intervals.

**FIGURE 10.**
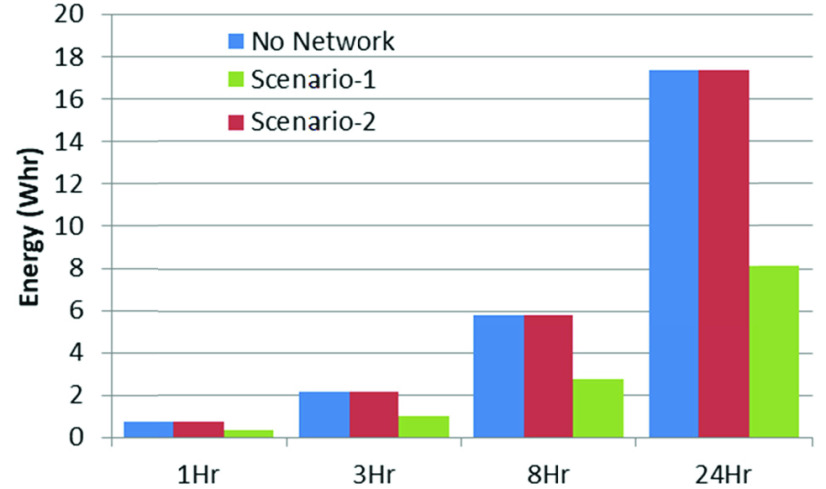
Scenario-specific hourly energy requirement.

Fig. 10 shows the energy requirement for various durations of daily operation using 15-minute transmission intervals. It also shows that local processing requires more than double the energy, compared with that required to send the unprocessed data over the wireless link.

**FIGURE 11.**
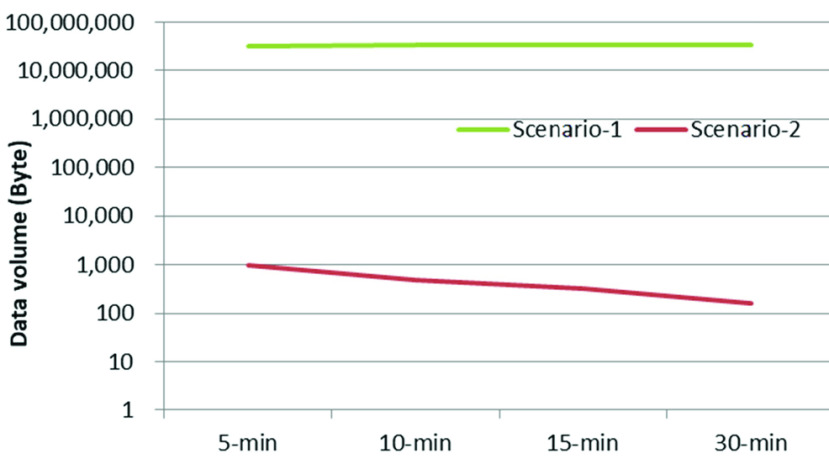
Scenario-specific hourly data volume for different transmission intervals.

**FIGURE 12.**
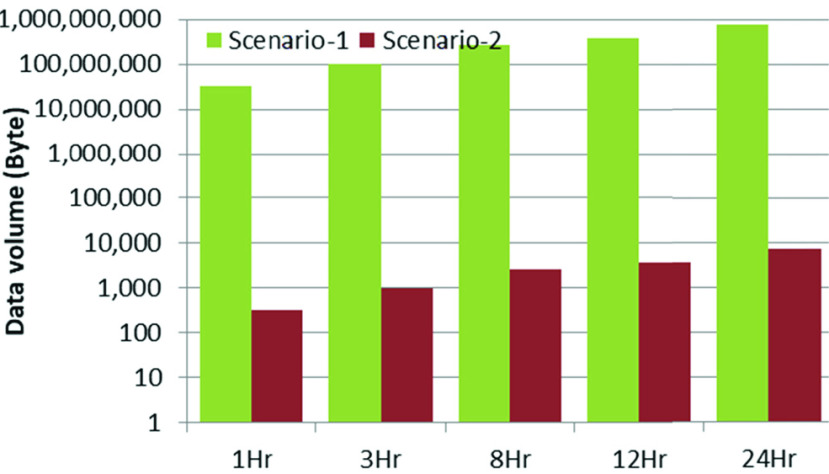
Scenario-specific data volume for different operation durations at 15-minute transmission intervals.

Since data acquisition and processing were carried out continuously, and the unprocessed data was sent to the network, hourly data volume remained the same for Scenario-1. However, it varied in Scenario-2 as only the processed data was sent. Fig. 11 shows the hourly data volume sent over the wireless links (both LoRa and Bluetooth) for different transmission intervals. Scenario-1 generated much higher data volume compared with Scenario-2 due to the transmission of unprocessed sensor data over the wireless link. Fig. 12 shows the data volume to be transferred over the wireless links at a transmission interval of 15 minutes for different durations of operation of the portable node.

After comparing both scenarios in terms of energy and bandwidth requirement, it can be seen that Scenario-1 is better fit for outdoor use, where the energy requirement could be a constraint and there is good quality wireless data connectivity, mainly in an urban area. On the other hand, Scenario-2 might be a better choice for rural areas with a lack of cellular network coverage. Therefore, the energy requirement can be reduced by increasing the data-transmission interval mainly for outdoor operation of the node.

Table 6 provides a summary of recent similar systems available to fight COVID-19 with the help of digital technology. As shown the proposed COVID-SAFE system presents a more complete IoT framework than others and can be used to control the infection after the pandemic. Many countries have implemented contact tracing apps, which are similar to the one shown in [21]. However, these apps merely trace a patient’s history and location, and notify users if anyone has contracted COVID-19 in the places they have recently visited. On the other hand, the proposed system provides hardware, sensors, and software (ML and mobile apps), which offer many other benefits, as shown in the table.

**TABLE 6:** Available Technologies for COVID-19 Pandemic

Ref	Description / Scope	Service offered					
		COVID symptoms	Hardware offered?	Diagnosis	Notification to person/ HA	Update real-time?	Remote monitoring
[1]	Overview of digital technology and COVID-19	No	No	No	No	No	No
[21]	Contact tracing, exposure notifications system by Apple and Google	No	Use smartphone	No	Yes, Yes	Yes	Yes
[22]	AI4COVID-19 – cough sample via an app	Yes (cough)	Use smartphone	Yes (cough samples)	Yes, No	Yes	No
[49]	AI-enabled CT for fast diagnosis of patients	No	No	Yes (CT scan)	No, Yes	No	No
[50]	nCapp diagnosis and treatment system	Yes (based on questionnaire only)	No (caregivers wear VR glass)	Yes (based on questionnaire only)	Yes, Yes	No	Yes
[51]	Smart-city network and AI-based universal data sharing standards	No	No	No	No	No	No
[52]	A plasmonic biosensor for COVID diagnosis	No	Yes	Yes (plasmon resonance)	No, Yes	No	No
[53]	Small wearables for social distancing	No	Developing kit	No	Yes	Yes	Yes
Our	COVID-SAFE – a complete IoT system	Yes (fever, cough, SpO_2_, HR, BMI, hotspots)	Yes (both smartphone and sensors to collect real-time data)	Yes (personal and regional data)	Yes, Yes	Yes	Yes

## V. Conclusion

In this article, an IoT framework is presented to monitor participants’ health conditions and notify them to maintain physical distancing. The proposed system integrates a wearable IoT node with a smartphone app, by which the IoT sensor node can collect a user’s health parameters, such as temperature and blood oxygen saturation, and the smartphone connects to the network to send the data to the server. The paper proposed a Radio Frequency (RF) distance-monitoring method which operates both for indoor and outdoor environments to notify users to maintain the physical distancing. Applying ML algorithms on body parameters makes it possible to monitor participant’s’ health conditions and to notify individuals in real time. A voice coughing-detector continually monitors the user’s voice and records the number and severity of coughing. The fog-based server is implemented to process received data from an IoT node using a cellular network or LoRa connection. In addition, locally processing the data makes it possible to use the IoT node in the environments without internet connectivity or fog-based networks. The system can assist participants in monitoring their daily activities and minimize the risk of exposure to the Coronavirus.
